# Systemic Sclerosis with Inflammatory Myositis: A Case Report

**DOI:** 10.31729/jnma.8298

**Published:** 2023-10-31

**Authors:** Suju Bhattarai, Jyoti Pudasaini, Ashutosh Sharma, Manjil Bharati, Narayan Dhakal

**Affiliations:** 1 Kathmandu Medical College and Teaching Hospital, Sinamangal, Kathmandu, Nepal; 2 Department of Internal Medicine, Shree Birendra Hospital, Chhauni, Kathmandu, Nepal

**Keywords:** *antibodies*, *case reports*, *inflammation*, *systemic sclerosis*

## Abstract

The overlap of systemic sclerosis and inflammatory myositis is a rare disorder which results in immune system activation and production of autoantibodies. We present a case of a 28-year-old female with complaints of generalized weakness, multiple joint pain, facial puffiness, and blackish discolouration of skin over the last 4 months. Blood investigations demonstrated autoantibodies positive for overlap syndrome. She was managed with hydroxychloroquine, mycophenolate mofetil and steroids. This case report highlights the importance of early diagnosis and treatment of this rare entity for better patient outcomes.

## INTRODUCTION

Systemic sclerosis (SSc) is a connective tissue disorder characterized by a triad of fibrosis, vasculopathy, and autoimmune manifestations in the form of inflammatory cell infiltrates and disease-specific diagnostic antibodies.^1^ Its pooled prevalence is 17.6 per 100000.^2^ Patients differ in terms of the severity and frequency of cutaneous and internal organ involvement. It can also be associated with numerous other autoimmune disorders including idiopathic inflammatory myopathy. This combination of conditions is linked to lower life expectancy and higher mortality.^3,4^ Hence, this case report emphasizes timely diagnosis and management of this rare condition to improve patient outcomes.

## CASE REPORT

A 28-year-old female presented to the outpatient department (OPD) with complaints of generalized weakness, multiple joint pain, facial puffiness, and blackish discolouration of skin for the last 4 months. She noticed swelling in her bilateral lower limbs and hands initially which gradually involved her face. She also had episodic Raynaud's phenomenon. She further complained of weight loss of 20 kg (previously weighed 74 kg and now weighed 54 kg) over 3 months. She also gave a history of two spontaneous abortions (one at 9 weeks and the other at 8 weeks).

On examination, she had hyperpigmentation of skin over both legs. She also had bilateral pitting oedema, hand swelling and facial puffiness. Her blood pressure was 100/60 mm of Hg and her pulse rate was 72 beats/min. Cardiovascular, neurological, abdominal and respiratory examinations were unremarkable. The patient also presented with sudden onset of cold fingers in association with sharply demarcated colour change of skin pallor during attack followed by bluish discoloration and with rewarming, ischemic changes usually took 15 to 20 min and upon recovery skin subsequently changed to pink colour. The above features were strongly indicative of Raynaud's phenomenon. Due to the other associated clinical features secondary Raynaud's phenomenon evaluation was warranted.

She was admitted to the medicine ward for further evaluation and a blood test was done. Her blood investigation revealed an increase in creatinine kinase (715 U/l). The rheumatoid arthritis (RA) factor was negative and anti-cyclic citrullinated peptide (anti-CCP) was 7.2. The patient was tested for autoantibodies and was anti-exosome (anti-PM-ScL) antibodies, antinuclear antibody by indirect immunofluorescence (ANA by IIF) and proliferating cell nuclear antigen (PCNA) positive. Antibodies for lupus anticoagulants were not present. Anti-cardiolipin, anti beta2 glycoprotein was negative. Nail fold capillaroscopy was performed and showed extensive avascular area, infarcts and late phase of sclerodermal pattern.

High-resolution computed tomography (HRCT) chest revealed fibrotic changes in both lungs with subtle glass ground changes bilaterally. Echocardiography was normal. Electromyography was normal. Magnetic resonance imaging (MRI) of the bilateral thigh showed evidence of subcutaneous oedema in the anterior thigh bilaterally.

Our patient had skin thickening of fingers, abnormal nail fold capillaries, Raynaud's phenomenon, systemic sclerosis-related auto-antibody (anti-scl-70) positive and features of interstitial lung disease in HRCT which suggested the diagnosis of systemic sclerosis. The patient also had an increased level of lactate dehydrogenase (LDH) and creatinine kinase (CK) along with features of subcutaneous oedema in the anterior thigh bilaterally in magnetic resonance which is suggestive of inflammatory myositis. Thus, our patient was diagnosed with a case of systemic sclerosis overlap syndrome.

The patient was started on hydroxychloroquine, mycophenolate mofetil and steroids. She was monitored for her symptoms. She had a gradual improvement in her symptoms. She was advised to follow up in OPD after 1 month. The steroid was gradually tapered off and she is presently on steroidsparing immunosuppressant under close monitoring.

## DISCUSSION

The combination of systemic sclerosis and idiopathic inflammatory myopathy is considered an overlap syndrome. The immune system activation in overlap syndrome results in the production of autoantibodies. Our patient had positive ANA by IIF which is associated with systemic sclerosis but also anti-PM-Scl antibodies, characteristic of SSc and inflammatory myositis overlap.^5^ Nailfold capillaroscopy, revealed a sclerodermal pattern which further helped to support the diagnosis of systemic sclerosis spectrum disease.

Up to 80% of SSc patients develop lung fibrosis, and 25% to 30% of individuals advance to interstitial lung disease.^6^ The HRCT of our patient showed fibrotic changes in the lungs which correlate with interstitial pulmonary involvement. It should be noted that lung involvement negatively impacts survival and quality of life which reinforces the need for close monitoring and early treatment. Additionally, the heart is frequently affected by the overlap of SSc and AIM and undiagnosed cardiovascular issues frequently result in mortality. Although electrocardiography or echocardiography done in our patient showed no cardiac involvement, these tests are not sensitive enough to detect heart fibrosis. Early stages of the disease are thought to be detectable by cardiac MRI.

The treatment for overlap syndrome is interdisciplinary and challenging. All potentially affected organs are checked and the treatment is based on disease-modifying and organ-specific drugs. In order to overcome the inflammatory process, corticosteroids were administered to our patient and she showed a good response. Various studies show the positive role of hydroxychloroquine and immunomodulatory medication, mycophenolate mofetil in the management of overlap syndrome.^7-9^ Our patient also showed marked improvement in symptoms after the use of these drugs.

The outcome of the case shows how important it is to diagnose and treat the patient at an earlier stage. For doctors to properly understand the experiences of their patients dealing with such diseases, it is imperative that they gain insights from this special perspective. This case intends to increase awareness of this uncommon condition, underline the importance of early discovery, stringent care, and the absolute necessity of developing safe and efficient treatments that are capable of enhancing outcomes and slowing the course of the disease.
